# Cortical folding of the preterm brain: a longitudinal analysis of extremely preterm born neonates using spectral matching

**DOI:** 10.1002/brb3.488

**Published:** 2016-05-17

**Authors:** Eliza Orasanu, Andrew Melbourne, Manuel Jorge Cardoso, Herve Lomabert, Giles S. Kendall, Nicola J. Robertson, Neil Marlow, Sebastien Ourselin

**Affiliations:** ^1^ Translational Imaging GroupCentre for Medical Image Computing (CMIC)University College LondonLondonUK; ^2^ INRIA ‐ Microsoft Research Joint CentrePalaiseauFrance; ^3^ Academic NeonatologyEGA UCL Institute for Women's HealthLondonUK

**Keywords:** Cortex, development, registration, shape analysis, spectra

## Abstract

**Introduction:**

Infants born extremely preterm (<28 weeks of gestation) are at risk of significant neurodevelopmental sequelae. In these infants birth coincides with a period of rapid brain growth and development, when the brain is also vulnerable to a range of insults. Mapping these changes is crucial for identifying potential biomarkers to predict early impairment.

**Methods:**

In this study we use surface‐based spectral matching techniques to find an intrasubject longitudinal surface correspondence between the white–grey matter boundary at 30 and 40 weeks equivalent gestational age in nine extremely preterm born infants.

**Results:**

Using the resulting surface correspondence, we identified regions that undergo more cortical folding of the white–grey matter boundary during the preterm period by looking at changes in well‐known curvature measures. We performed Hotelling T^2^ statistics to evaluate the significance of our findings.

**Discussion:**

The prefrontal and temporal lobes exhibit most development during the preterm period, especially in the left hemisphere. Such correspondences are a promising result as longitudinal measurements of change in cortical folding could provide insightful information about the mechanical properties of the underlying tissue and may be useful in inferring changes during growth and development in this vulnerable period.

## Introduction

Infants that were born extremely preterm are at a higher risk of developing cognitive and neurologic impairment, despite advances in neonatal intensive care (Marlow et al. 2005). During the last 10 weeks of pregnancy, major changes occur in the appearance and connectivity of the fetal brain. During this relatively short period of time, the cortex develops from a lissencephalic state to a very folded one and dramatically increases in volume and surface area (Kapellou et al. 2006). Following premature birth the structural development of the brain takes place under the altered conditions of the extrauterine environment. Abnormal gyrification patterns have been associated with cognitive‐behavioral deficits among subjects with developmental language disorder, autism, or dyslexia (Zhang et al. 2010).

Recently, there has been much interest in understanding changes in altered brain development during the preterm period (Volpe 2009). Boardman et al. (2010) identified a common phenotype in 66 of 80 preterm infants scanned at term‐equivalent age, consisting of diffuse white matter injury and focal tissue loss localized in several brain regions. Ball et al. (2012) acquired T_1_‐, T_2_‐weighted and diffusion MRI from 71 preterm infants scanned at term‐equivalent age and combined deformation‐based morphometry, Tract‐Based Spatial Statistics and tissue segmentation were used to study the effect of prematurity on regional tissue volume and microstructure. They concluded that reduced volume of the thalamus implied lower cortical volume and decreased volume in the frontal and temporal lobes. Melbourne et al. (2013) scanned 92 infants at term‐equivalent age and demonstrated that the cortical sulcation ratio of local white matter regions is significantly correlated with the gestational age at birth in these infants. Furthermore, they found that the cortical sulcation ratio (the ratio between the number of surface points with positive shape index and the total number of surface points) varies spatially over the cortical surface, whereas the fractional anisotropy of white matter regions varies according to location. Moeskops et al. (2015). investigated the cortical development in a cohort of 85 preterm infants scanned at 30 and 40 weeks postmenstrual age, by looking at the longitudinal global changes such as volume, surface area, global mean curvature, thickness, and gyrification index. They proved that there were larger global changes in the occipital lobes and that the gyrification index and global mean curvature decrease with abnormality score, whereas thickness increases. All these studies demonstrate that preterm birth has a high impact on cortical development. However, it is not known how gyrification in the preterm brain changes over time (Kesler et al. 2006) and accurate measurements of the brain during this early postnatal period may yield predictive biomarkers of neurological outcome (Boardman et al. 2010). Furthermore, most of these studies mentioned above (Boardman et al. 2010; Ball et al. 2012; Melbourne et al. 2013) are not longitudinal and do not look at individual local changes in the preterm born infants.

The human brain evolves significantly during the last trimester of pregnancy. The development of the prefrontal cortex, as well as the temporal lobe, takes place mostly during this time period, later than the parietal and occipital cortex, making it possible to study their development in more detail using longitudinal data of infants aged between 30 and 40 weeks equivalent gestational age. Due to the developmental timing, both these regions may be more affected by preterm birth and thus finding feasible biomarkers to predict future impairment is of great interest.

The prefrontal cortex (PFC) is situated in the anterior part of the frontal lobes of the brain, and it is thought to play an important role in cognitive control, executive function, and habituation (Miller and Cohen 2001). Because of its anatomical connections with the cortical and subcortical centers, important for movement control, the PFC plays a role in coordinating motor function (Diamond 2000). Thus, accurate measurements in this region, in particular its shape change, might be predictive of early delays in motor control. The superior frontal gyrus of the PFC becomes defined by 25 gestational weeks (GW) (Chi et al. 1977). The inferior frontal sulcus is visible by 28 GW, followed by the delineation of the middle and inferior gyri (Chi et al. 1977). All three main regions show secondary gyri at about 32 GW, whereas tertiary gyri are distinctive by 40 GW (Chi et al. 1977).

The temporal lobe plays a crucial role in the formation of explicit long‐term memory and appears to be an area of increased vulnerability in the preterm brain as abnormalities in its morphology may contribute to learning difficulties (Kesler et al. 2006). Most of the temporal lobe development takes place after 30 weeks of gestation. By this time, the middle temporal and inferior temporal gyrus can be distinguished, whereas the superior temporal gyrus begins to be recognizable already from about 23 weeks of gestation (Chi et al. 1977). A right‐left asymmetry can be noticed in the temporal lobe as the right transverse temporal gyrus develops at approximately 31 GW, whereas the left transverse temporal gyrus develops 1 or 2 weeks later (Chi et al. 1977). The secondary sulci of the superior temporal gyri are visible at 34–35 GW, whereas the secondary gyri of the transverse temporal gyrus appear after 36 GW (Chi et al. 1977).

The parietal lobe plays an important role for sensory integration and visual attention (Hanson et al. 2013) and the occipital lobe is the visual processing center of the human brain, containing most of the visual cortex (Wang et al. 2012). These two lobes have a different time frame of development and the gyri and sulci corresponding to these regions develop before 28 weeks of gestation (Chi et al. 1977), thus they are not as affected by preterm birth as the prefrontal cortex and temporal lobe.

In order to study the local cortical differences at different time points, a spatial correspondence has to be defined between them. Matching of cortical surfaces is a challenging process and nonlinear registration methods only take into account local deformations. As our surfaces have very different levels of folding, we need a method that will consider the global changes that take place. Most methods of cortical surface matching that address this problem are based on either optimizing flows, such as LDDMM (Beg et al. 2005), or on inflating surfaces to a common template which is usually a sphere, such as FreeSurfer (Fischl et al. 1999) and Spherical Demons (Yeo et al. 2010). Spectral graph matches shapes in the spectral domain, where two near‐isometric shapes, with identical geodesic distances between points, have identical spectral representations (Lombaert et al. 2013; Shi et al. 2014). This method represents thus a promising tool for matching shapes with different levels of folding, but similar representations in the spectral domain.

In this study we use Joint‐Spectral Matching techniques as described by Lombaert et al. (2013), initialized with a Coherent Point Drift algorithm (Myronenko and Song 2010), to find a longitudinal correspondence between preterm and term‐equivalent white–grey matter boundary of the brain of nine extremely preterm‐born infants. This is extremely challenging as the brain undergoes significant chances in cortical folding, shape, surface area, and volume. We then investigate whether matching different brain regions independently from one another influences the matching result. Using the determined point correspondence, we look at which regions undergo more cortical folding of the white–grey matter boundary during the preterm period by using well‐known curvature measures. We performed Hotelling T^2^ statistics to evaluate the significance of our findings. The purpose of this study was to demonstrate that spectral matching can be used in the challenging problem of matching brains with different levels of folding, like in the case of the preterm infants, and to use this result look at gyrification regions. This work builds upon our previous work (Orasanu et al., 2015b), in which we focused only on the matching and development of the prefrontal cortex.

## Methods

### Subjects

Subjects were recruited for this study as part of the University College Hospital (UCH) Preterm Development Project. The study was approved by the local research ethics committee and informed parental consent was obtained for all infants. For this study, we had a very strict inclusion criteria and we included infants that did not present abnormal cerebral ultrasound, had good‐quality MR images, and who did undergo both scans.

We used nine infants with mean gestational age at birth (GAB) of 26.8 ± 1.5 weeks. The infants were scanned first shortly after birth, at average EGA of 31.6 ± 1.1 weeks and then at term‐equivalent age, at average EGA of 41.7 ± 2.9 weeks. None of the infants was growth restricted. All mothers were on prenatal steroids, but none of the infants were given postnatal steroids. Characteristics of these infants are summarized in Table 1.

**Table 1:** Description of preterm‐born infants subjects used in the study

Subject identifier	Estimated gestational age at birth (weeks + days)	Birth weight (g)	EGA at scan 1 (weeks + days)	Quality control (QC) of scan 1	EGA at scan 2 (weeks + days)	QC of scan 2
a	26 + 1	784	33 + 1	Slight T1 motion	40 + 1	Good
b	25 + 1	730	31 + 3	Good	42	Good
c	25 + 1	760	31	Good	42	Good
d	27 + 1	1038	30 + 6	Slight T1 motion	46 + 2	Good
e	27 + 1	880	29 + 6	Good	46 + 2	Good
f	26 + 2	940	31 + 6	Good	40 + 2	Good
g	29 + 1	1250	31 + 1	Good	38 + 3	Good
h	29 + 2	1020	32 + 4	Slight T1 motion	38 + 6	Good
i	26	856	33	Good	41	Good

### MR acquisition

The data were acquired on a Philips Achieva 3T (Amsterdam, Netherlands) MRI machine. The infants were imaged in a MR‐compatible incubator after feeding, when spontaneously asleep, without any sedation.

The T_1_‐weighted data used a 3D MP‐RAGE at a resolution of 0.82 × 0.82 × 0.5 mm at TR/TE = 17/4.6 msec, acquisition duration 462 sec.

### Image preprocessing

The images underwent bias field correction using the N3 algorithm of FreeSurfer (Sled et al. 1998; RRID:SCR_001847), a process that was necessary in order to minimize the registration error induced by intensity nonuniformity as a result of the MR acquisition. Brain masks of the intracranial volume of the 40‐week EGA images were resampled from a publicly available neonate brain atlas described in (Kuklisova‐Murgasova et al. 2011) after a nonrigid registration between each image and the atlas template. Brain masks for the early 30‐week EGA images were resampled from their corresponding 40‐week EGA mask after affine alignment. All masks were checked and manually corrected to exclude any nonbrain tissue that can generate mislabeled voxels (Xue et al. 2007) in the subsequent segmentation using ITK‐SNAP (Yushkevich et al. 2006, RRID:SCR_002010).

### Infant brain segmentation and parcellation

**Figure 1:**
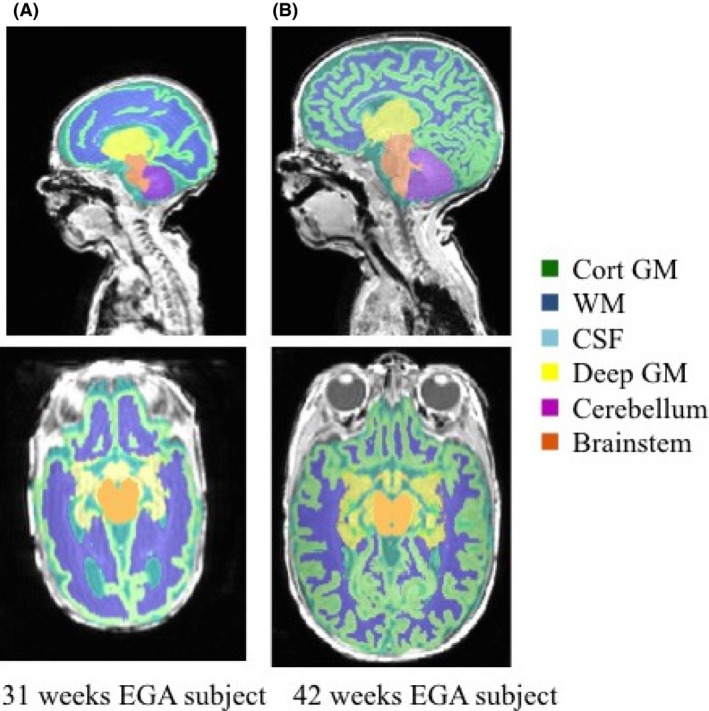
Infant brain segmentation into cortical grey matter (GM), white matter (WM), cerebrospinal fluid (CSF), deep grey matter (dGM), cerebellum, and brainstem of infant *c* for two different time points: 31 and 42 weeks EGA, respectively.

We segmented each infant brain into six different tissue classes: grey matter, white matter, cerebrospinal fluid (CSF), deep grey matter, cerebellum, and brainstem, using a preterm‐specific Expectation‐Maximization (EM) segmentation with prior relaxation, AdaPT, as described by (Cardoso et al. 2013; Melbourne et al. 2013). Anatomical priors were resampled in the same way as the brain masks from the same publicly available neonate brain atlas (Kuklisova‐Murgasova et al. 2011) after affine alignment with brain mask. An example of the brain segmentation for subject **c** is shown in Figure 1.

To separate the prefrontal cortex, temporal lobe, occipital lobe, and parietal lobe, we used a multicontrast human neonatal brain atlas (Oishi et al. 2011) registered to the space of the early and late scans using nonrigid registration (Modat et al. 2010). The corresponding regions were grouped by hemisphere: left and right.

As in this study we are looking at the cortical folding, of particular interest is the white–grey matter boundary. Thus, the white matter segmentation was combined with the region parcellations to investigate this boundary. Any segmentation errors were corrected by morphological operations (largest connected component and filling of the holes), as well as manual corrections when needed, to ensure a topologically correct surface. The processed left and right white matter region segmentations were used to create smooth triangle‐based meshes of each surface using ITK‐SNAP (Yushkevich et al. 2006, RRID:SCR_002010).

### Spectral matching

Spectral matching methods have been used recently by several authors with different applications (Lombaert et al. 2013; Orasanu et al. 2015a; Wright et al. 2015) to find correspondences in the spectral domain. In summary, for any surface model *S* = {*V, E*}, with *V* the set of vertices *V* = (*x*_*1*_ *,…,x*_*N*_) and *E* the edges of the surfaces, we can define the weighted adjacency matrix *W* in terms of node affinities with $w_{ij}\;=\;e_{ij}/\left\|x_{1}-x_{j}\right\|$, where *e*_*ij*_ = 1 if nodes *i* and *j* are connected and 0 otherwise. The general Laplacian operator L is given by $L=D^{-1}\left(D-W\right)$, where *D* is the degree matrix, a diagonal matrix with $d_{ii}\;=\;\sum_{j}W_{ij}$.

The graph decomposition is given by diagonalizing the general graph Laplacian $L=U\Lambda U^{-1}$, which produces the eigenvalues $\Lambda \;=\;(\lambda_{0},\lambda_{1},\ldots ,\lambda_{N})$ and eigenfunctions *U* = (*U*_*0*_ *, U*_*1*_ *,…,U*_*N*_). The eigenvectors or eigenmodes represent the fundamental modes of vibrations of the surface, and, respectively, describe increasing complexity of its geometric features, from coarse to fine scales. The first eigenfunction *U*_0_ is discarded as it is the null vector.

The spectral representation of the shape *S* can be denoted by using the first *k* spectral components as the *k*‐dimensional embedding of the shape where each node *i* has the spectral coordinates defined as (*U*_1*i*_ *, U*_2*i*_ *,…,U*_*ki*_), which is a row of the truncated matrix *U*. A point‐wise correspondence map *c* can then be established with pairs of closest points, defined by the shortest Euclidean distance, between the spectral representation of the surfaces *S*_1_ and *S*_2_.

### Joint‐spectral matching of cortical surfaces

A rigid Coherent Point Drift (CPD) algorithm was used to find an initial correspondence for the intrasubject cortical regions at the two different time points (Myronenko and Song 2010). CPD was preferable to the Feature Oriented Correspondence using Spectral Regularization (FOCUSR) used by Lombaert et al. (2012), as we optimize for a rigid transformation, rather than a nonrigid one, resulting in a simpler and more efficient optimization.

After an initial correspondence was computed, Joint‐Spectral Matching (JSM) (Lombaert et al. 2013) was used to find the correspondence for the intrasubject white matter surface at 30‐week EGA and term‐equivalent age for each subject. JSM builds a dual‐layered graph, where layers correspond to the cortical surface of each subject, and correspondence links, found from the initial intrasubject matching, that connect both layers. This joint approach of connected surfaces produces a set of shared eigenmodes and, thus, a shared parameterization of the surfaces. The meshes we want to match are very different in size, shape, and morphology; however, their representation in the spectral domain has a less complex topology, making the two surfaces comparable and easier to map a correspondence between them using a nearest‐neighbor search (Lombaert et al. 2013).

**Figure 2:**
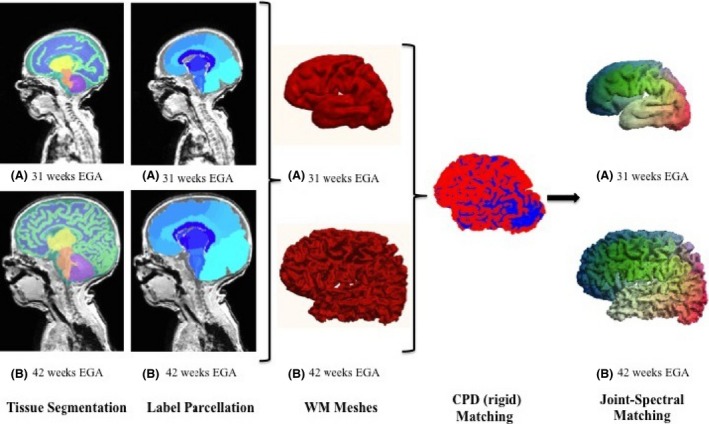
The spectral matching algorithm. After preterm‐specific tissue segmentation and labeling the different regions, meshes of the grey–white matter boundary are defined for the 31‐ and 42‐week EGA time points of infant c. After a rigid Coherent Point Drift initialization (blue and red correspond to the two now overlapping meshes), a point‐wise correspondence is defined by Joint‐Spectral Matching (spectral matching color‐coded anterior‐posterior).

The algorithm process is summarized in Figure 2.

### Consistency of surface matching

We compared the obtained correspondence of the intrasubject PFC, temporal, occipital, and parietal meshes, respectively, with a whole‐brain white matter intrasubject correspondence in all subjects, in order to check the cortical surface matching consistency and how it is influenced by the quality of the meshes. For each vertex that existed in both the whole‐brain and regional meshes at scan 1, the mismatch was computed by calculating the Euclidean distance of the corresponding vertices, defined by the spectral matching, in the regional‐ and whole‐brain mesh at scan 2.

### Measurement of longitudinal development

To quantify the early cortical folding we computed the principal curvatures *k*_1_ and *k*_2_ for each vertex of the surface mesh from which we derived the mean and Gaussian curvatures, $M\;=\;\frac{1}{2}\left(k_{1}+k_{2}\right)$ and G = *k*1 · *k*_2_, respectively.

Additionally, we computed the bending energy *E* (Joshi and Sèquin 2007), which is intuitively dependent on the local surface area change. The change in the bending energy, over time and over area, describes the energy needed to deform that area, which may be able to provide localized information about the mechanical properties of the underlying tissue:

$$\frac{\partial E}{\partial t}\;=\;\frac{\partial }{\partial t}\int \left(k_{1}^{2}+k_{2}^{2}\right)dA$$

To investigate the longitudinal brain development for each subject, we mapped the 40 weeks surfaces to the 30 weeks surfaces using JSM and computed the change in the surface parameters at each vertex of the mesh. The longitudinal change in the parameters (*M*,* G*,* E*) can be computed by measuring the change between the parameter value at any given point on one surface and its corresponding point on the other surface, while correcting for volume and surface area differences.

To have comparable measures of curvature for all brain meshes at different EGA, the values have to be corrected for enclosed mesh volume. The volume of each white matter mesh was computed as the sum of all voxels from each segmentation, which was then multiplied by the voxel dimension provided by the scanner. The values for the mean and Gaussian curvatures were then corrected for brain volume by dividing each value by the cube root of the ratio of the subject's white matter mesh volume and mean white mesh matter volume of the 30‐ and 40‐week EGA (Awate et al. 2010).

The difference in total curvature (sum of squared principal curvatures) was corrected by taking into account the local area change between the 30 and 40 weeks subjects to obtain the bending energy. We estimated the surface area of the meshes by calculating the sum of the triangles’ areas. By using the correspondence between the areas, a local measurement of surface area change can be defined (Orasanu et al. 2015b). Then the total curvature value at each vertex in the 40‐week mesh was divided by the local area change at that vertex and then mapped into the space of the 30‐week mesh, where it was multiplied by the local area change in the 30‐week mesh.

### Longitudinal differences: group analysis

We performed a group analysis to look at the significance of the folding changes during the preterm period. We selected one reference infant and for each time point and hemisphere we mapped, using JSM initialized by CPD, all other infants, by time point and hemisphere (Shakeri et al. 2014). The mappings were used to create mean shapes for the two time points. Morphological changes between the two time points were investigated after another step of JSM with a CPD initialization. Differences in shape represent differences in cortical folding: the more different two shapes are, the more difference there is in the degree of folding. We used the Hotelling T^2^ two sample metric to derive a local group shape difference metric and local statistical *P*‐values for all corresponding points (Styner et al. 2007). Group‐wise shape analysis using spectral matching has previously been shown to give reliable results (Shakeri et al. 2014).

## Results

### Joint‐spectral matching correspondence

**Figure 3:**
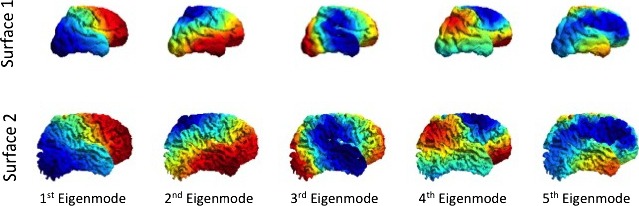
The first five spectral modes of infant *c* for two different time points: 31 and 42 weeks EGA, respectively. Although the meshes are quite different in cartesian space, they have similar representations in the spectral domain.

We found a good qualitative point correspondence between all 9 pairs of 30‐ and 40‐week white–grey matter boundary meshes of both left and right hemisphere, PFC, temporal, occipital, and parietal lobe of all subjects. The first five spectral modes (Figure 3) for infant ***c*** are shown below and describe variation of shape in the spectral domain, features that were optimized to find a surface correspondence. We notice that although the two shapes are different in Cartesian coordinates, they are very similar in the spectral domain, as low‐frequency spatial features between infants at different time points are similar and advanced secondary and tertiary gyrification is represented by high‐frequency information (Tallinen et al. 2016).

### Consistency of surface matching

The surface matching obtained by individually matching the prefrontal cortex and temporal lobe and matching the entire brain did not have significant variations. The PFC mean average distance mismatch for all infants was 1.46 ± 0.96 mm for the left hemisphere and 1.91 ± 1.28 mm for the right hemisphere. The temporal lobe mean average distance mismatch among all infants was 2.63 ± 1.40 mm and 2.78 ± 1.34 mm for the left and right hemispheres, respectively. Furthermore, the occipital lobe had a mean average distance mismatch among all infants of 2.54 ± 1.52 mm and 2.47 ± 1.52 mm for the left and right hemispheres, respectively, whereas for the parietal lobes the values were 2.63 ± 1.53 mm and 2.85 ± 1.59 mm for the left and right hemispheres, respectively.

**Figure 4:**
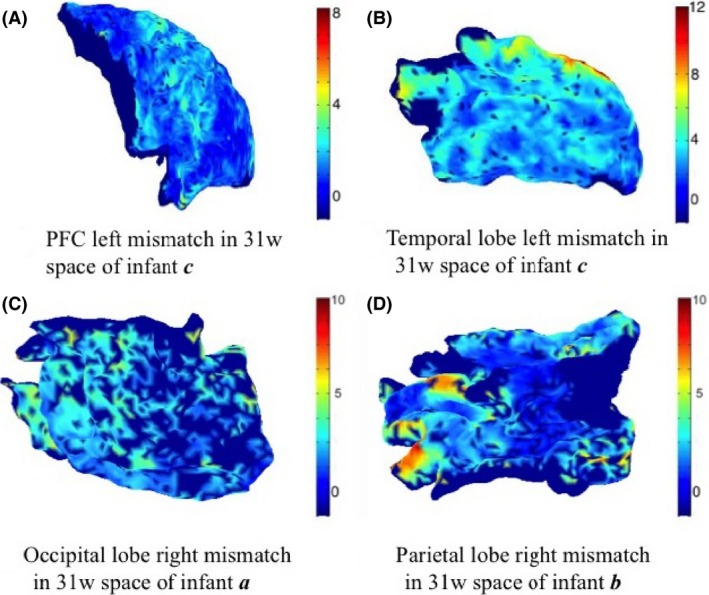
Example maps of mismatching variation between the whole‐brain matching and regional spectral matching correspondences (units are in mm). Vertices that are not represented in both individual and whole‐brain meshes were attributed a value of −1 (represented with dark blue).

Example maps of these variations in all regions are shown in Figure 4.

### Longitudinal brain development over the preterm period

#### Whole brain

**Figure 5:**
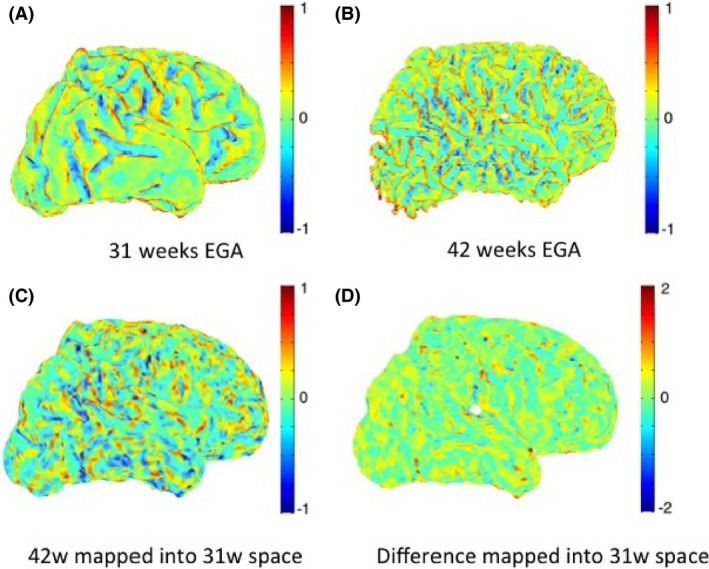
Maps of mean curvature of whole‐brain white–grey matter boundary (left) for infant c scanned at 31 weeks (A) and 42 weeks (B) EGA. Positive values (red/yellow) represent gyri (convex structures) and negative values (blue) represent sulci (concave structures). The Joint‐Spectral Matching correspondence allows us to map mean curvatures from 42‐week to 31‐week space (C) and compute the changes in mean curvature between these two time points in 31 weeks space (D).

**Figure 6:**
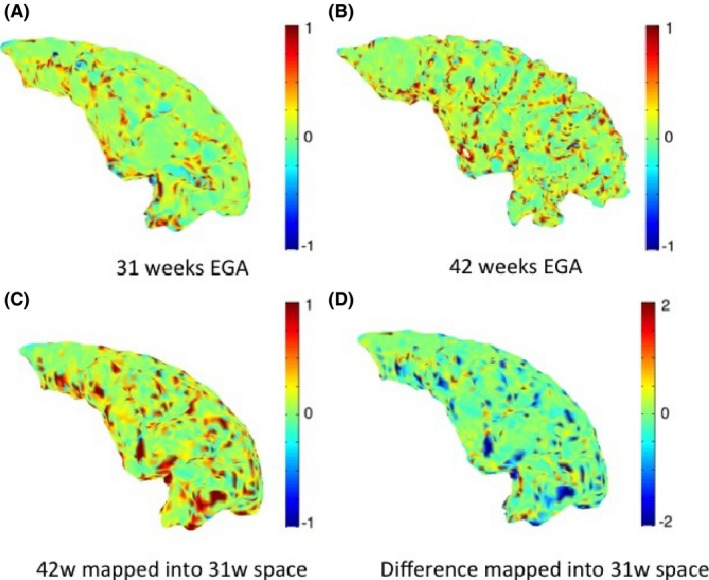
Maps of Gaussian curvature of the grey–white matter boundary of the prefrontal cortex (left) for infant c scanned at 31 weeks (A) and 42 weeks (B) EGA. It can be seen that the Gaussian curvature is more sensitive to geometric errors of the meshes and noisier than the mean curvature maps.

Figure 5 shows an example of maps of mean curvature for the white–grey matter boundary of subject ***c*** scanned at 31 and 42 weeks EGA. The maps of mean curvature change in 31 weeks space (Fig. 5D) show the apparent locations where the secondary and tertiary sulci and gyri will be formed, as well as how the primary sulci and gyri will develop. The regions that indeed exhibit the most folding are the prefrontal cortex and the temporal lobes. We note that the Gaussian curvature is very sensitive to the geometric errors on the white–grey matter interface and thus not as reliable and useful a feature for cortical surface analysis as the mean curvature (Fig. 6).

#### Prefrontal cortex

**Figure 7:**
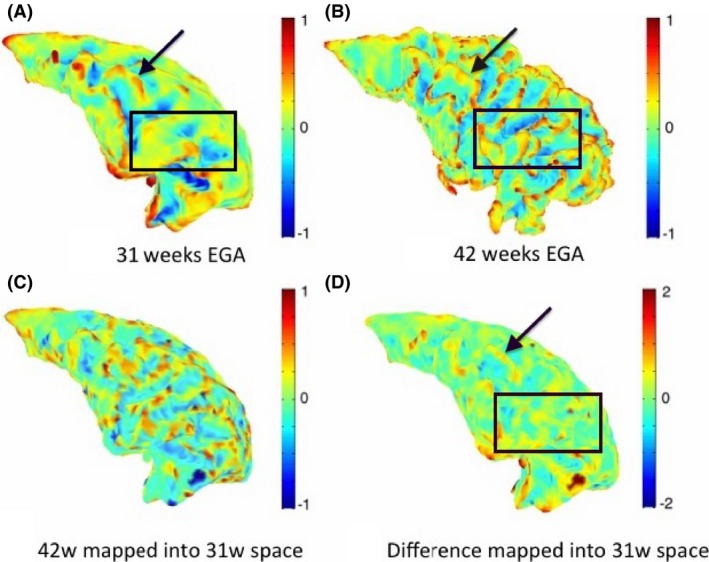
Maps of mean curvature of the grey–white matter boundary of the prefrontal cortex (left) for infant c scanned at 31 weeks (A) and 42 weeks (B) EGA. Positive values (red/yellow) represent gyri (convex structures) and negative values (blue) represent sulci (concave structures). The Joint‐Spectral Matching correspondence allows us to map mean curvatures from 42 week to 31 week space (C) and compute the changes in mean curvature between these two time points in 31 weeks space (D). The difference map of the mean curvature over the preterm period indicates the further development of several primary gyri and sulci, like the middle frontal gyrus (indicated by the black arrow), as well as regions where secondary and tertiary sulci will emerge (black square).

Figure 7 shows an example of maps of mean curvature for the prefrontal cortex white–grey matter boundary of subject ***c*** scanned at 31 and 42 weeks EGA. The maps of mean curvature change in 31‐week space (Fig. 7D) show the apparent locations where the secondary and tertiary sulci and gyri will be formed especially in the middle and inferior region, as well as how the primary sulci and gyri, like the middle frontal gyrus, will develop. These changes are commensurate with Chi et al. (1977).

**Figure 8:**
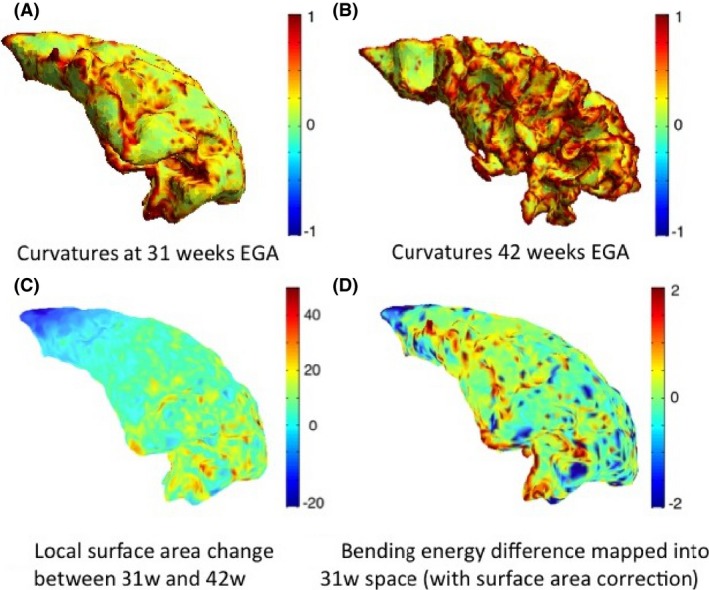
Total curvature of the white–grey matter boundary of the prefrontal cortex (left) shown for infant *c* at 31 weeks (A) and 42 weeks (B) EGA, local surface area change between the two time points (C), and computed bending energy (D). Positive values (red) in the bending energy represent regions of gyrification, and negative values (blue) represent regions of sulcation.

As describe above in Measurement of longitudinal development, the bending energy depends on both the total curvature difference and the change in local surface area. The inferior prefrontal cortex undergoes more local surface area change compared to the superior prefrontal cortex, which undergoes almost no change (Fig. 8C). This is consistent with the observation that superior PFC development and folding is largely complete before 31 weeks (Chi et al. 1977).

#### Temporal lobe

**Figure 9:**
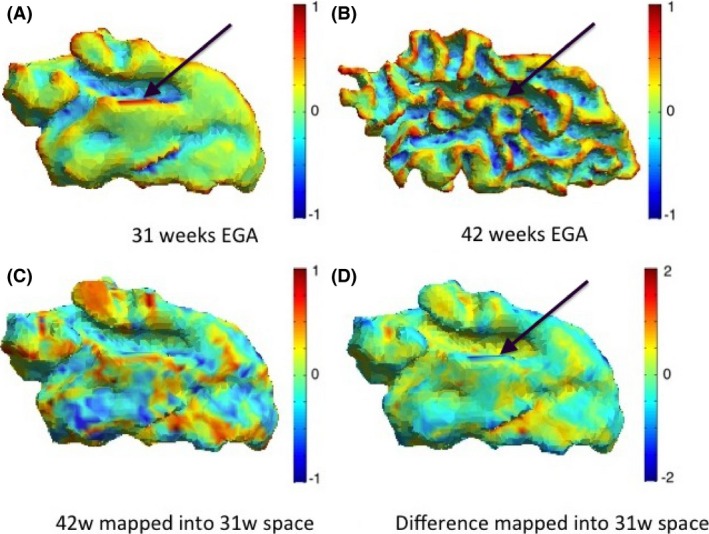
Maps of mean curvature of the grey–white matter boundary of the temporal lobe (left) for infant *c* scanned at 31 weeks (A) and 42 weeks (B) EGA. Positive values (red/yellow) represent gyri (convex structures) and negative values (blue) represent sulci (concave structures). The Joint‐Spectral Matching correspondence allows us to map mean curvatures from 42‐week to 31‐week space (C) and compute the changes in mean curvature between these two time points in 31 weeks space (D). The black arrow indicates the transverse temporal gyrus, which becomes definite around 31 weeks of gestation and therefore all other secondary and tertiary gyri surrounding it will emerge during the preterm period.

Similarly, Figure 9 shows an example of maps of mean curvature for the left temporal lobe white–grey matter boundary for the same subject ***c*** scanned at 31 and 42 weeks EGA. The maps of mean curvature change in 31 weeks space indicate the likely further development of the primary gyri and sulci and emergence of most of the secondary and tertiary gyri and sulci. Probably, the most striking is the evolution of gyri and sulci around the transverse temporal gyrus, situated across the ‘middle’ part of the temporal lobe, as this gyrus becomes definite much later than other primary gyri, around 31 weeks of gestation. Although this gyrus is visible in the early scan, all secondary and tertiary gyri surrounding it emerge during the preterm period. These changes are, again, commensurate with Chi et al. (1977).

**Figure 10:**
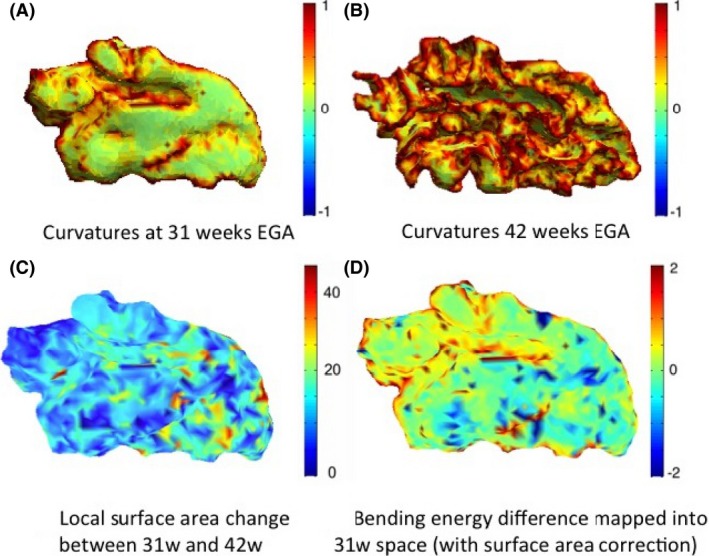
Total curvature of the white–grey matter boundary of the temporal lobe (left) shown for infant c at 31 weeks (A) and 42 weeks (B) EGA, local surface area change between the two time points (C), and computed bending energy (D). Positive values (red) in the bending energy represent regions of gyrification, and negative values (blue) represent regions of sulcation.

The map of local surface area change (Fig. 10C) shows that the frontal region of the temporal lobe, especially around the transversal gyrus expands the most. The maps describing the bending energy required for the cortical folding development of the temporal lobe in the preterm period may be linked to the mechanical deformation required for the folding of the cortex.

#### Occipital and parietal lobes

**Figure 11:**
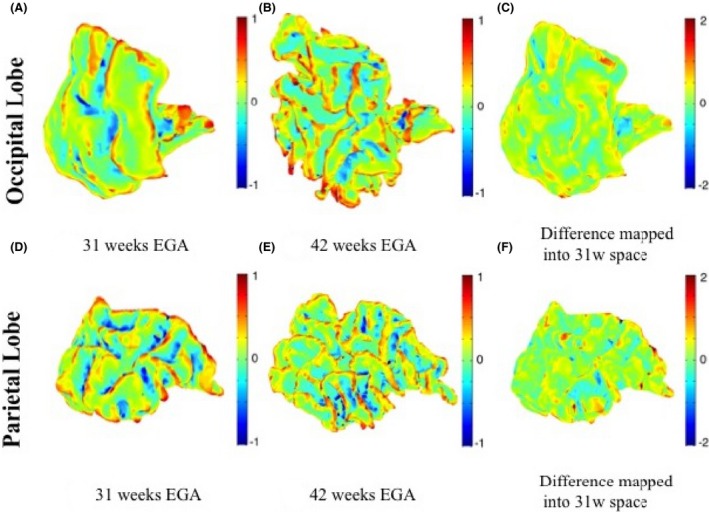
Maps of mean curvature of the grey–white matter boundary of the left occipital lobe (upper row) and left parietal lobe (bottom row) for infant c scanned at 31 weeks (A and D) and 42 weeks (B and E) EGA. Positive values (red/yellow) represent gyri (convex structures) and negative values (blue) represent sulci (concave structures). The Joint‐Spectral Matching correspondence allows us to map mean curvatures from 42‐week to 31‐week space and compute the changes in mean curvature between these two time points in 31 weeks space (C and F). It can be noticed that most differences (c and f) appear on already existing gyri and sulci as most of them are already formed by the time of the first scan.

Similarly, Figure 11 shows an example of maps of mean curvature for the occipital and parietal lobes white–grey matter boundary for the same subject *c* scanned at 31 and 42 weeks EGA. The maps of mean curvature change in 31 weeks space (Fig. 11C and F) do show that there does not seem to be any appearance of new gyri or sulci during the preterm period for these regions and only the existing features continue to develop, as described by (Chi et al. 1977).

#### Longitudinal brain development over the preterm period in all infants

We investigated the change in surface area as function of equivalent gestational age. Across all infants, the surface area for all individual regions and both hemisphere increases linearly with equivalent gestational age (*R* ^2^ > 0.88). The rates of change in surface area per week for each region, by hemisphere, are summarized in Table 2. We also report that the volume increase as a function of GA is not linear (*R* ^2^ < 0.85).

**Table 2:** Surface area increase as a function of equivalent gestational age in nine preterm‐born infants, scanned both at early (31.6 weeks EGA) and term‐equivalent age time points (41.7 weeks EGA)

	Linear increase in left hemisphere white matter (cm^2^/week)	Linear increase in right hemisphere white matter (cm^2^/week)
Whole brain	26.23 (*R* ^2^ = 0.96)	25.79 (*R* ^2^ = 0.97)
Prefrontal cortex	6.00 (*R* ^2^ = 0.98)	6.21 (*R* ^2^ = 0.94)
Temporal lobe	3.44 (*R* ^2^ = 0.97)	3.20 (*R* ^2^ = 0.94)
Occipital lobe	3.86 (*R* ^2^ = 0.90)	3.77 (*R* ^2^ = 0.88)
Parietal lobe	5.80 (*R* ^2^ = 0.97)	5.59 (*R* ^2^ = 0.96)

The mean curvature maps appeared to be the most reliable curvature measure, as gyrification and sulcation patterns can be clearly observed in each subject for both prefrontal cortex and temporal lobe. Therefore, to examine the variation in developmental pattern between 30 and 40 GW, we show the change in mean curvature maps for all seven infants across all regions investigated.

**Figure 12:**
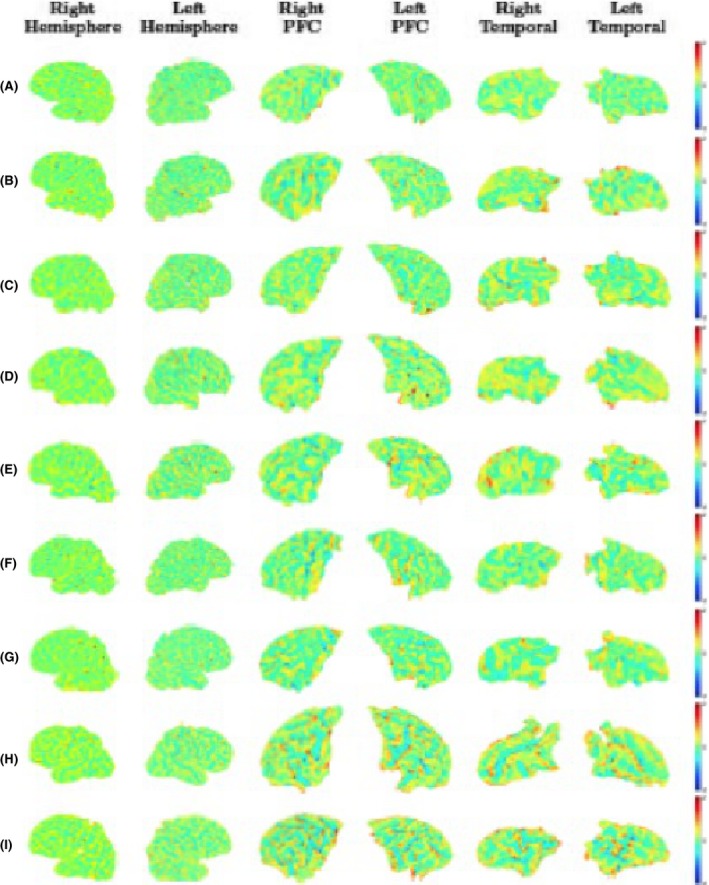
Changes in the intrasubject mean curvature mapped into the earlier scan using Joint‐Spectral Matching for all nine infants. For each infant (A–I) we show the mean curvature changes in the regions we looked at: right hemisphere (first column), left hemisphere (second column), prefrontal cortex right hemisphere (third column), prefrontal cortex left hemisphere (fourth column), temporal lobe right hemisphere (fifth column), and temporal lobe left hemisphere (sixth column). Positive values (red/yellow) represent gyri (convex structures) and negative values (blue) represent sulci (concave structures).

Figure 12 shows development of primary sulci and gyri and the emergence of secondary and tertiary gyri and sulci mapped onto the meshes from the early scan of each infant. Results are consistent with infant ***c***. Variation in these images is introduced by three main factors: (1) intersubject variability; (2) the gestational age of the infant; and (3) the temporal gap between the two scans (the infants were not scanned at the exact same two time points). For example, it is noticeable how infant ***e*** (scanned around 29 and 46 weeks EGA) shows more changes in mean curvature in both prefrontal cortex and temporal lobe than infant ***g*** (scanned around 31 and 38 weeks EGA). There seems to be an asymmetry between the left and right hemisphere in both prefrontal cortex and temporal lobes, the latter one showing less change during the preterm period, consistent with other observations (Chi et al. 1977).

**Figure 13:**
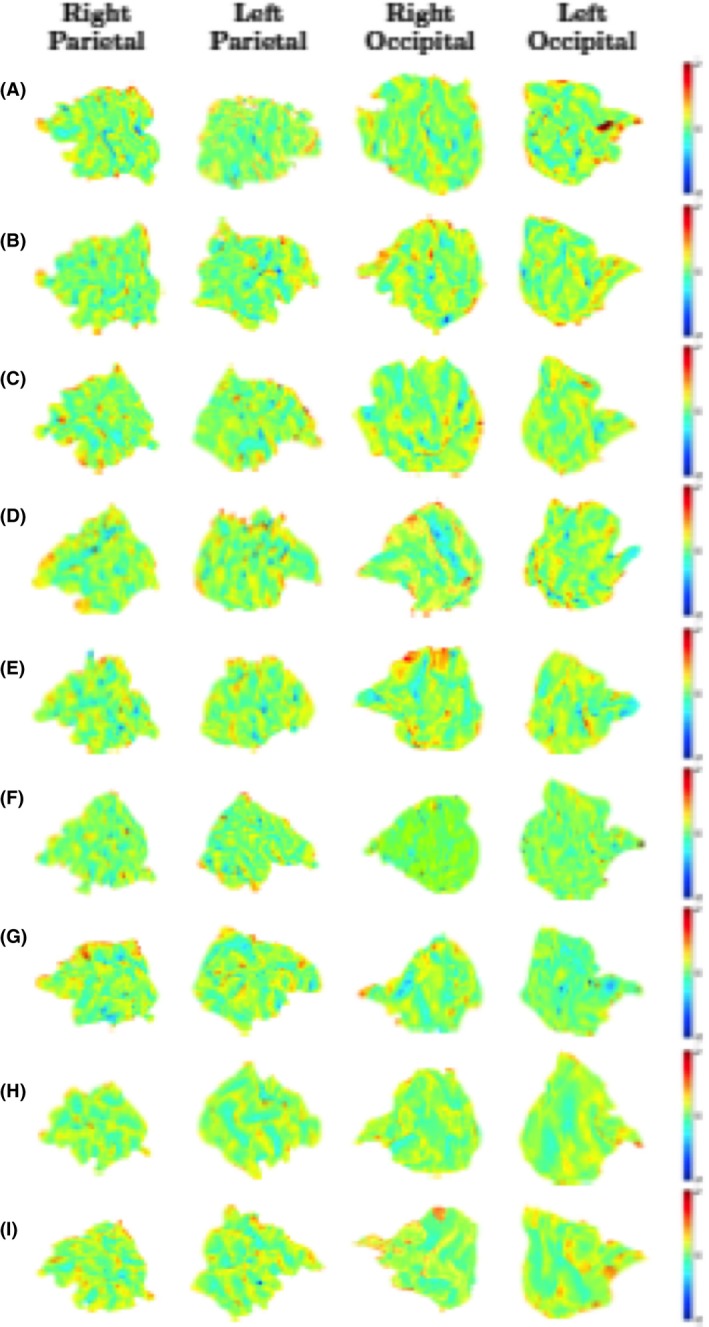
Changes in the intrasubject mean curvature mapped into the earlier scan using Joint‐Spectral Matching for all nine infants. For each infant (A–I) we show the mean curvature changes in the regions we looked at: right parietal lobe (first column), left parietal lobe (second column), right occipital lobe (third column), and left occipital lobe (fourth column). Positive values (red/yellow) represent gyri (convex structures) and negative values (blue) represent sulci (concave structures).

The developmental changes in all infants of the mean curvature of the parietal and occipital lobe are shown in Figure 13. It can be seen, as expected, that there are less changes in these two lobes than in the prefrontal cortex or temporal lobe, and that most of these changes occur on already existent gyri and sulci, with almost no new appearances of new gyri and sulci.

#### Longitudinal differences: group‐wise analysis

**Figure 14:**
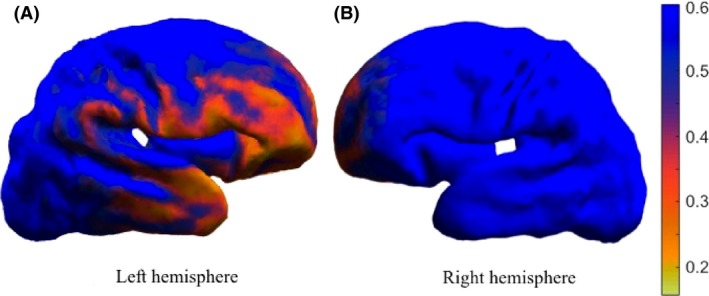
Statistical maps of longitudinal differences during the preterm period in nine infants of the left and right hemisphere. The *P*‐values are described by the color bar.

Figure 14 shows the corresponding *P*‐values of the differences between the two time points for each vertex, mapped on the mean early scan. We noticed that the regions with statistically significant difference between the two time points (*P*‐value < 0.5) are the left and right prefrontal cortex (the middle and lower regions) and the left temporal lobe (the region surrounding the transversal temporal gyrus), the same regions where we noticed differences in the longitudinal data for individual infants.

## Discussions

We have used joint‐spectral matching techniques to find an intrasubject correspondence between the white–grey matter boundary of nine extremely preterm infants scanned shortly after birth, around 30 weeks EGA, and at term‐equivalent age, around 40 weeks EGA. We then verified if using smaller meshes for individual regions rather than the whole brain affects gives consistent correspondences. Finally, we investigated the change in well‐known measures of surface curvature such as the mean curvature to look at the regions that undergo the most cortical folding during this crucial period, as well as the significance of these changes.

Although the number of subjects used might seem relatively small, it is actually quite large if we take into account the difficulty of acquiring such novel data, as extremely preterm birth is a relatively rare event and health of the subjects is often an issue. This sample is enough to test our methods.

The intrasubject white matter meshes are very different in size, shape, and cortical folding, resulting in the failure of nonlinear registration techniques. The spectral shape representations, however, denoted by their first five spectral components or modes of vibration, are very similar (see Fig. 3); thus, JSM provides a very good qualitative correspondence. JSM was initialized using a rigid CPD correspondence, as it provides a more robust alignment for meshes that are so different, compared to FOCUSR. This novel method has much potential for the assessment of development in the preterm brain.

The maps of surface matching variation as well as the average inconsistency between the different spectral matches of the same regions show that the correspondences determined from the spectral matching are quite consistent. The variations in the prefrontal cortex matching are smaller than those of the other lobes. The larger matching inconsistencies are usually situated at the boundaries of the region's mesh, as, when finding a correspondence for a smaller region of the brain, there is no additional information about the shape of the surrounding neighboring regions to further guide the matching.

We investigated the cortical folding by looking at the change in mean curvature in the prefrontal cortex, temporal, occipital, and parietal lobe. The uniqueness of the application motivated us to choose well‐known measures of curvature and cast these as measures of longitudinal change in the individual. We also examined the bending energy because it may provide information about the underlying tissue deformation and can be interpreted directly as a measurement of change in the infant folding pattern. Looking at the maps of changes in mean curvature for all selected regions in all infants, we did notice how the prefrontal cortex and temporal lobe develop more new gyri and sulci than the occipital and parietal lobe during this preterm period.

Finally, we performed a group‐wise analysis where we created a mean shape of the early and late time point of all infants. By performing Hotelling T^2^ statistics, we noticed that regions of significant shape difference and gyrification, and thus folding differences, are the left temporal lobe, especially the regions around the transverse temporal gyrus, and the left and right prefrontal cortex, the inferior and middle regions (*P*‐value < 0.5). These results are consistent with our initial expectations that these regions develop mostly in the third trimester of pregnancy; hence they might be affected by preterm birth. Previous studies have found reduction in white and grey matter volumes of temporal and frontal lobes in other preterm cohorts (Fraello et al. 2011; Engelhardt et al. 2015), usually correlated with neurological deficits, poor executive function, and language deficits. Furthermore, we noticed a left‐right asymmetry, with the left hemisphere suffering more changes than the right hemisphere. The right temporal lobe does not show statistically significant changes; hence it may not be affected by preterm birth as it is also known to develop 2 weeks earlier than the left temporal lobe. Dubois et al. (2008) also found a right‐before‐left development of the superior temporal sulcus during the preterm period.

The main limitation of our study is that, although qualitatively we obtained a very good correspondence for the intrasubject prefrontal cortex and temporal lobe, it is very difficult to validate this cortical matching, as the data are novel and there is no proper way of establishing a ground truth. Manual validation by a trained neuroanatomist could be used for validation, but it would be restricted to the most obvious and easy to capture anatomical landmarks. Another limitation can also be the age range of our subjects, however, the size of our meshes were corrected for when looking at the changes, as described in Measurement of longitudinal development.

Generating accurate correspondences between the intrasubject brain white matter at multiple time points enables us to measure the longitudinal changes that take place in this region in preterm infants. These measures may contribute to developing early biomarkers for predicting executive and motor development. This type of research might begin to illuminate the debate on the mechanical role of tissue growth on the observed cortical folding pattern, information that is only measurable in fetal and neonatal cohorts of this type.

## Conclusion

In this study, we used joint‐spectral matching techniques to find a correspondence for the longitudinal white matter in extremely preterm‐born neonates. The correspondence was then used to look at the longitudinal development of the preterm brain and specific regions, like the prefrontal cortex, temporal, parietal, and occipital lobe, which depicted the emergence of secondary and tertiary gyri and sulci. We hope that these type of measures, together with the neurological outcome of the infants, may contribute to early biomarkers for predicting executive and motor development.

## Conflict of Interest

The authors declare no conflicts of interest.

## Supporting information


**Appendix S1** We present here the computed total volume and surface area of the white matter of each hemisphere and then each particular region for all nine subjects, plotted as a function of EGA.
