# Cortical folding of the preterm brain: a longitudinal analysis of extremely preterm born neonates using spectral matching

**DOI:** 10.1002/brb3.585

**Published:** 2016-09-22

**Authors:** Eliza Orasanu, Andrew Melbourne, Manuel Jorge Cardoso, Herve Lombaert, Giles S. Kendall, Nicola J. Robertson, Neil Marlow, Sebastien Ourselin

In Orasanu et al. (2016), the last name of the fourth author was spelled incorrectly as Herve Lomabert. The author's correct name should be Herve Lombaert.
