# Supplementary material for: Cortical folding of the preterm brain: a longitudinal analysis of extremely preterm born neonates using spectral matching
Source: Brain Behav. 2016 May 17;6(8):e00488. doi: 10.1002/brb3.488 (PMC4873564; doi:10.1002/brb3.488)
Supplement: Supplementary file 1 — Appendix S1 We present here the computed total volume and surface area of the white matter of each hemisphere and then each particular region for all nine subjects, plotted as a function of EGA. [file BRB3-6-e00488-s001.docx]

# Appendix

We present here the computed total volume and surface area of the white matter of each hemisphere and then each particular region for all 9 subjects, plotted as a function of EGA. We used these values for correction of the meshes statistics. We also report here the linear increase values of the surface areas as a function of EGA in Table 2. The volume increase as a function of GA is not linear ($R^{2}<0.85$).


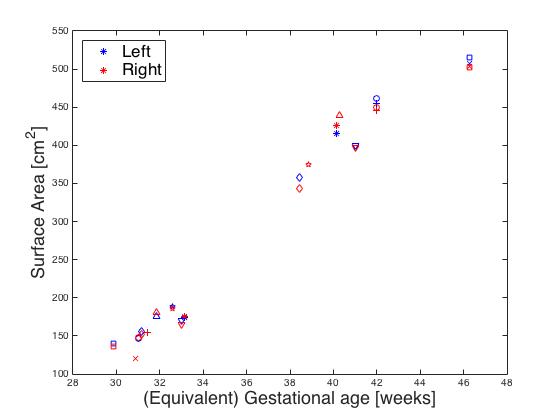

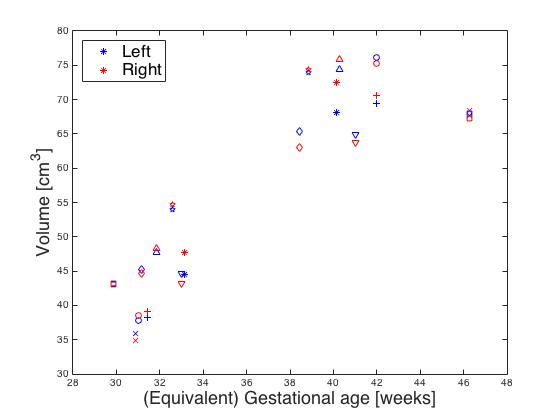


Figure 1. Surface area and volume change for all 9 subjects of the left (blue) and right (red) hemispheres as a function of GA. Subjects have unique marker identifiers: a - asterisk, b - addition sign, c - circle, d - cross, e - square, f - triangle, g - diamond, h – star, i – upside-down triangle.


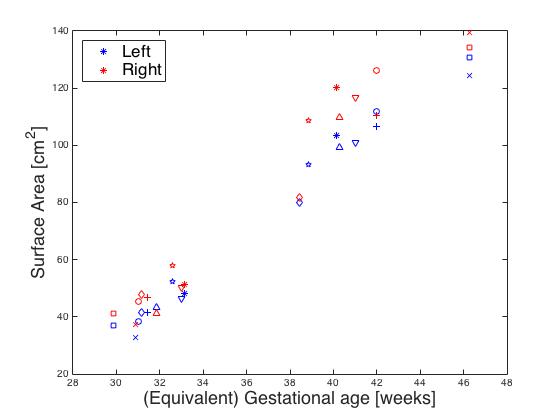

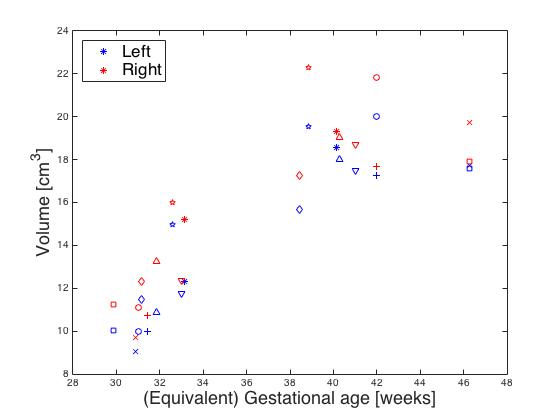


Figure 2. Surface area and volume change for all 9 subjects of the left (blue) and right (red) prefrontal cortex as a function of GA. Subjects have unique marker identifiers: a - asterisk, b - addition sign, c - circle, d - cross, e - square, f - triangle, g - diamond, h – star, i – upside-down triangle.


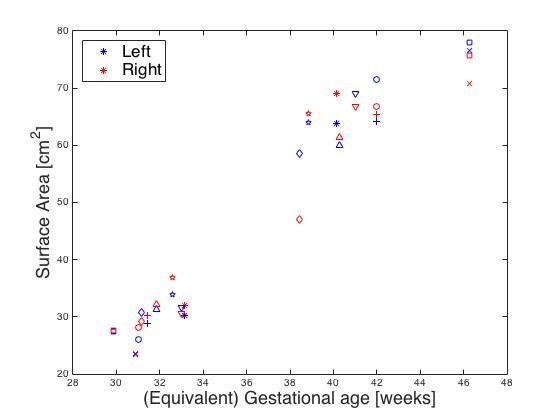

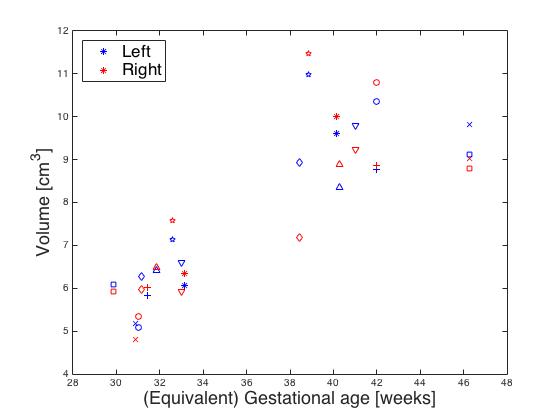


Figure 3. Surface area and volume change for all 9 subjects of the left (blue) and right (red) temporal lobe as a function of GA. Subjects have unique marker identifiers: a - asterisk, b - addition sign, c - circle, d - cross, e - square, f - triangle, g - diamond, h – star, i – upside-down triangle.


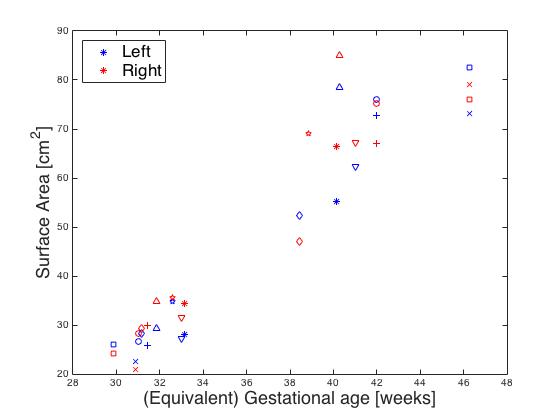

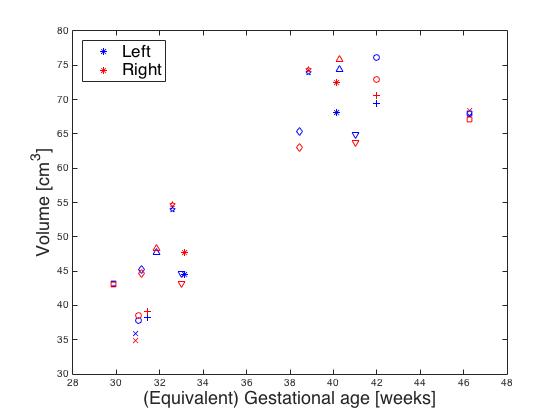


Figure 4. Surface area and volume change for all 9 subjects of the left (blue) and right (red) occipital lobe as a function of GA. Subjects have unique marker identifiers: a - asterisk, b - addition sign, c - circle, d - cross, e - square, f - triangle, g - diamond, h – star, i – upside-down triangle.


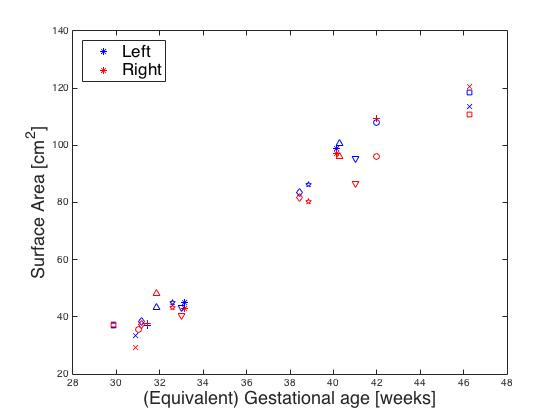

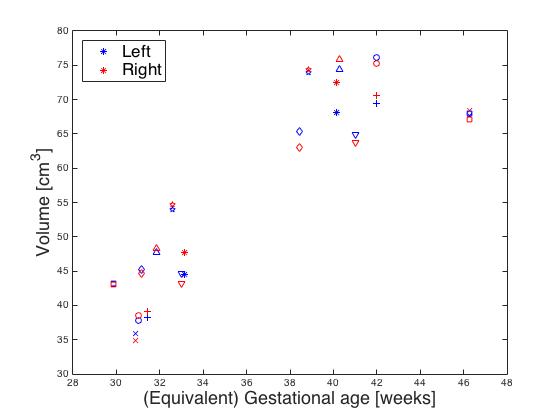


Figure 5. Surface area and volume change for all 9 subjects of the left (blue) and right (red) parietal lobe as a function of GA. Subjects have unique marker identifiers: a - asterisk, b - addition sign, c - circle, d - cross, e - square, f - triangle, g - diamond, h – star, i – upside-down triangle.
